# Epigastric pain syndrome accompanying pancreatic enzyme abnormalities was overlapped with early chronic pancreatitis using endosonography

**DOI:** 10.3164/jcbn.17-41

**Published:** 2017-08-18

**Authors:** Satomi Hashimoto, Seiji Futagami, Hiroshi Yamawaki, Keiko Kaneko, Yasuhiro Kodaka, Mako Wakabayashi, Noriko Sakasegawa, Shuhei Agawa, Kazutoshi Higuchi, Teppei Akimoto, Nobue Ueki, Tetsuro Kawagoe, Hitomi Sato, Katsuhisa Nakatsuka, Kaya Gudis, Chiaki Kawamoto, Takashi Akamizu, Choitsu Sakamoto, Katsuhiko Iwakiri

**Affiliations:** 1 Division of Gastroenterology, Nippon Medical School Nippon Medical School, 1-1-5 Sendagi, Bunkyo-ku, Tokyo 113-8602, Japan; 2 The First Department of Medicine, Wakayama Medical Unversity, 811-1 Kimiidera, Wakayama 641-8509, Japan

**Keywords:** functional dyspepsia, epigastric pain syndrome, early chronic pancreatitis, endosonography, chronic pancreatitis

## Abstract

There was not available data about the overlap between functional dyspepsia (FD) and pancreatic diseases. We aimed to determine whether epigastric pain syndrome (EPS) accompanying with pancreatic enzyme abnormalities were associated with early chronic pancreatitis proposed by Japan Pancreas Society (JPS) using endosonography. We enrolled 99 consecutive patients presenting with typical symptoms of FD, including patients with postprandial distress syndrome (PDS) (*n* = 59), EPS with pancreatic enzyme abnormalities (*n* = 41) and EPS without pancreatic enzyme abnormalities (*n* = 42) based on Rome III criteria. Gastric motility was evaluated using the ^13^C-acetate breath test. Early chronic pancreatitis was detected by endosonography and graded from 0 to 7. The ratio of female patients among EPS patients (34/41) with pancreatic enzyme abnormalities was significantly (*p* = 0.0018) higher than the ratio of female EPS patients (20/42) without it. Postprandial abdominal distention and physical component summary (PCS) scores in EPS patients with pancreatic enzyme abnormalities were significantly disturbed compared to those in EPS patients without it. Interestingly, AUC_5_ and AUC_15_ values (24.85 ± 1.31 and 56.11 ± 2.51, respectively) in EPS patients with pancreatic enzyme abnormalities were also significantly (*p* = 0.002 and *p* = 0.001, respectively) increased compared to those (19.75 ± 1.01 and 47.02 ± 1.99, respectively) in EPS patients without it. Overall, 64% of EPS patients with pancreatic enzyme abnormalities were diagnosed by endosonography as having concomitant early chronic pancreatitis proposed by JPS. Further studies are warranted to clarify how EPS patients with pancreatic enzyme abnormalities were associated with early chronic pancreatitis proposed by JPS.

## Introduction

According to the Rome III classification criteria, the major symptoms of functional dyspepsia (FD) consist of bothersome postprandial fullness, early satiety, epigastralgia and epigastric burning.^(1)^ Thus, visceral hypersensitivity in response to distention,^(2)^ impaired meal accommodation^(3)^ and delayed gastric emptying have frequently been demonstrated in patients diagnosed with FD.^(4–6)^ Furthermore, the involvement of several other mechanisms has also been suggested, including duodenal hypersensitivity to the luminal contents, small bowel dysmotility, *Helicobacter pylori* (*H. pylori*) infection,^(7)^ psychological disturbances^(8)^ and central nervous system disorders.^(9,10)^ Since FD patients, especially epigastric pain syndrome (EPS) patients, can be classified into heterogenic subgroups, the use of proton pump inhibitors in the treatment of EPS is controversial.^(11–15)^ Previous study has reported an association between FD and pancreatic dysfunction. Ashizawa *et al.*^(16)^ have reported that camostat mesilate, a common treatment for pancreatitis, is a significantly more effective therapy than famotidine in the treatment of epigastralgia. However, there are no data available that can explain how FD associates with pancreatic disease.

Chronic pancreatitis was reported to be one of the causes of dyspepsia,^(17)^ in the absence of abnormal findings on laboratory tests, ultrasonography, computed tomography and upper endoscopy. Anderson *et al.*^(18)^ have reported that 35% of patients with dyspepsia suffer from pancreatic dysfunction. In addition, 27% of subjects with functional dyspepsia have been reported to have pancreatic juice abnormalities consistent with chronic pancreatitis.^(19)^ Moreover, Sahai *et al.*^(17)^ have also reported that dyspepsia may be an atypical presentation of pancreatic disease using endoscopic ultrasonography (EUS). EUS provides a practical means to look for subtle pancreatic abnormalities that may be missed by conventional imaging studies such as abdominal ultrasound sonography (US), computed tomography (CT) and magnetic resonance imaging (MRI). In particular, studies have shown that EUS has the sensitivity to discover the slight pathological changes associated with chronic pancreatitis.^(20,21)^

In Japan, to hinder the initial phase of chronic pancreatitis from advancing into chronic pancreatitis, new strategies for addressing chronic pancreatitis have been proposed as early chronic pancreatitis.^(22)^ In this study, we aimed to determine whether epigastric pain syndrome accompanying with pancreatic enzyme abnormalities associates with early chronic pancreatitis using endosonography and compared clinical symptoms and gastric emptying between epigastric pain syndrome with pancreatic enzyme abnormalities and without it.

## Materials and Methods

### Patients

This study enrolled 99 consecutive patients presenting with typical symptoms of FD, including 59 patients with postprandial distress syndrome (PDS) and 83 patients with EPS, after upper gastrointestinal endoscopy, abdominal ultrasonography and abdominal computed tomography. Patients were diagnosed according to the Rome III criteria.^(23)^ Exclusion criteria included severe heart disease, renal or pulmonary failure, liver cirrhosis, severe systemic illness and history of malignant disease. *H. pylori* infection was determined by both the ^13^C-urea breath test and by measurement of anti-*H. pylori* antibody. We measured amylase, lipase, trypsin, PLA2 and elastase-1 in the sera of the patients with FD. Written informed consent was obtained from all subjects prior to undergoing upper gastrointestinal endoscopy and abdominal ultrasonography for evaluation of dyspeptic symptoms. The study protocol was approved by the Ethics Review Committee of Nippon Medical School Hospital.

### Clinical symptoms

Clinical symptoms of FD were evaluated according to the Rome III criteria.^(23)^ In this study, we enrolled PDS patients without abdominal pain or epigastric burning. FD symptoms including epigastric pain, epigastric burning, postprandial fullness and early satiety, and satisfaction with treatment, were assessed by visual analogue scale (VAS scale: 0–10; 0 = absent, and 10 = maximal).^(24–27)^ Clinical symptoms were evaluated with the Gastrointestinal Symptom Rating Scale (GSRS).^(28)^ We used the mean score of the GSRS and the 15 GI symptoms of the GSRS for the evaluation of dyspeptic symptoms.

### Assessment of endosonography

**Fig. 1:**
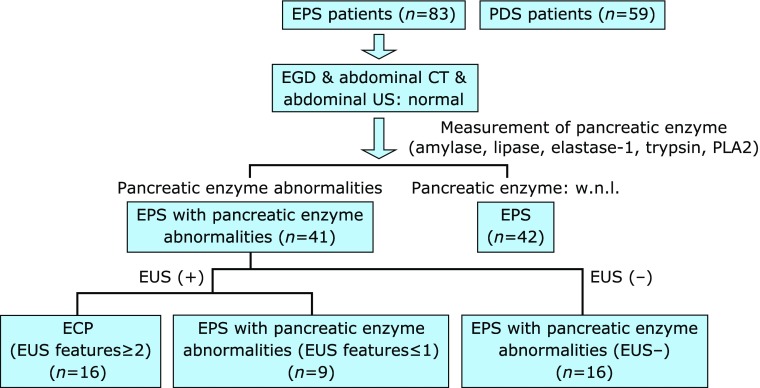
Flowchart of this study. ECP, early chronic pancreatitis.

An Olympus EUS-UCT 260 convex scanning endosonography (Olympus America, Melville, NY) at 7.5 MHz was used to perform EUS under conscious sedation. Endosonographic parenchymal or ductal abnormalities were recorded based on new guideline.^(22)^ Diagnosis of early chronic pancreatitis can be made by imaging findings as more than 2 EUS features of above seven items (1. Lobularity with honeycombing, 2. Lobularity without honeycombing, 3. hyperechoic foci without shadowing, 4. stranding, 5. cysts, 6. dilated side branches, 7. hyperechoic MPD margin) and clinical findings as two or more than items among repeated attacks of upper abdominal pain, abnormalities in blood/urine pancreatic enzymes, exocrine pancreatic dysfunction and persistent drinking history (80 g/day) (Fig. 1).

### Pittsburgh Sleep Quality Index (PSQI)

Sleep quality and sleep duration were evaluated by a Japanese version of the Pittsburgh Sleep Quality Index (PSQI) questionnaire.^(29)^ Higher scores indicate poorer sleep.^(29,30)^ A cut-off score >5.5 has a sensitivity of 80.0–85.7% for various patient groups, and a specificity of 86.6% for control subjects in the Japanese version of the PSQI.^(30)^

### Health-related quality of life (HRQOL)

The Social Functioning-8 (SF-8) test was used to measure health-related quality of life (HRQOL) according to the Manual of the SF-8 Japanese Version.^(31)^ A score <50 thus indicates impaired quality of life (QOL), with lower scores considered to indicate greater damage to QOL.

### State-trait anxiety inventory (STAI)

The STAI is a well-validated 40-item self-reported questionnaire to evaluate degree of anxiety.^(32)^ State of anxiety reflects a “transitory emotional state or condition of the human organism that is characterized by subjective, consciously perceived feelings of tension and apprehension, and heightened autonomic nervous system activity.” State of anxiety may fluctuate over time and can vary in intensity. In contrast, trait of anxiety denotes “relatively stable individual differences in anxiety proneness”.

### Measurement of gastric emptying

The liquid test meal consisted of 100 mg of ^13^C-acetate dissolved in 200 ml of a liquid meal (Racol, 1 ml/kcal; Otsuka Pharmacia Company, Tokyo, Japan). We used an Integrated Software Solutions program to calculate the half gastric emptying time (T_1/2_) and the lag phase (Tmax; min) as the point of maximum gastric emptying according to Hellmig *et al.*^(33)^ The half gastric emptying time (T_1/2_) represents the time when 50% of the initial gastric content was emptied. Tmax value greater than 60 min, representing the mean Tmax in healthy volunteers plus SD, was defined to represent relative disturbances in gastric emptying according to the diagnostic criteria of the Japan Society of Smooth Muscle Research and our study.^(7,34)^

### Data analysis

The time plot of pulmonary [^13^CO_2_] excretion (%dose/h) was fitted to the function:

(%dose/h) = m × k × β × e^−kt^ × (1 − e^−kt^)^β − 1^

where “m” is the cumulative [^13^CO_2_] recovery at the infinite time, “t” is in hours and “k” and “β” are regression-estimated constants.

(Cumulative %dose) = m × (1 − e^^−kt^^)β

AUC_5__ _= m × (1 − e^−k × 0.08^)β [T: 5 min = 0.08 h]

AUC_15_ = m × (1 − e^−k × 0.25^)β [T: 15 min = 0.25 h]

(AUC: are under the curve)

We determined the area under the curve at 5 min (AUC_5_) and AUC_15_ values as markers of the early phase of gastric emptying based on previous studies.^(25,35–38)^ AUC_5_ values of >17.4 and AUC_15_ values of >39.6, representing the mean AUC value of healthy volunteers plus 2SD, were defined to represent disturbances in the early phase of gastric emptying.

### Statistical analysis

For statistical evaluation of group data, Students’ *t* test for paired data and analysis of variance (ANOVA) for multiple comparisons were followed by Scheffe’s *F* test. Mann-Whitney *U* test was used for analysis of categorical data. Data analyses were performed by using a standard software package (SPSS ver. 13.0, Chicago, IL). A *p* value of less than 0.05 was statistically significant.

## Results

### Characteristics of PDS patients, EPS patients with pancreatic enzyme abnormalities and EPS patients without pancreatic enzyme abnormalities

**Fig. 2:**
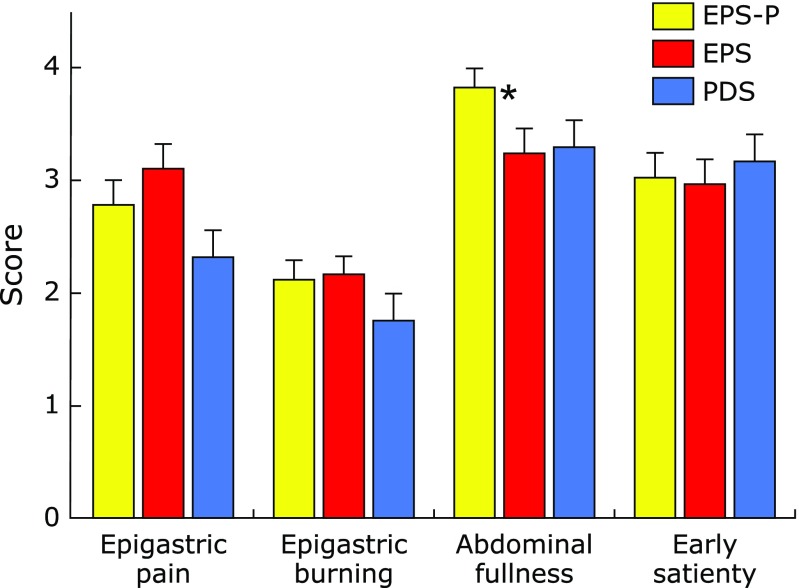
Comparison of FD symptoms among EPS patients with pancreatic enzyme abnormalities, EPS patients without it and PDS patients. Score of postprandial abdominal fullness in EPS patients with pancreatic enzyme abnormalities was significantly higher compared to that in EPS patients without it. *p = 0.041, vs EPS patients without pancreatic enzyme abnormalities.

Age, *H. pylori* infection, and total GSRS did not differ statistically among 59 PDS patients, 41 EPS patients with pancreatic enzyme abnormalities and 42 EPS patients without pancreatic enzyme abnormalities (Table 1). There was one PDS patient (1/59) with pancreatic enzyme abnormalities in our study. In addition, the ratio (34/41) of female patients in EPS patients with pancreatic enzyme abnormalities was significantly (*p* = 0.0018) higher than the ratio (20/42) of those in EPS patients without it (Table 1). Moreover, scores for postprandial abdominal distention in EPS patients with pancreatic enzyme abnormalities were significantly (*p* = 0.041) higher compared to those of EPS patients without it (Fig. 2). There were no significant differences in epigastric pain, epigastric burning and early satiety among three groups (Fig. 2). We measured a panel of pancreatic enzymes including amylase, elastase-1, lipase, trypsin and PLA2 in the sera of the patients with FD. We found that the measurement of trypsin in the sera of the patients with FD had a positivity rate of 76.2%.

### Comparison of SF-8, global PSQI and STAI scores among PDS patients, EPS patients with pancreatic enzyme abnormalities and EPS patients without pancreatic enzyme abnormalities

Physical component summary (PCS) scores in EPS patients with pancreatic enzyme abnormalities had significantly (*p* = 0.024 and *p* = 0.004, respectively) lower compared to those of EPS patients without pancreatic enzyme abnormalities or PDS patients (Table 2). In addition, the mental component summary (MCS) score of EPS patients with pancreatic enzyme abnormalities was significantly (*p* = 0.02) lower compared to that of PDS patients (Table 2). However, the MCS score of EPS patients with pancreatic enzyme abnormalities did not differ significantly from that of EPS patients without it. Global PSQI scores in the EPS patients with pancreatic enzyme abnormalities were significantly (*p* = 0.005 and *p* = 0.016, respectively) lower compared to those in the EPS patients without pancreatic enzyme abnormalities and PDS patients. In addition, there were no significant difference in STAI-state/-trait scores among three groups (Table 2).

### Comparison of gastric motility between EPS patients with pancreatic enzyme abnormalities and EPS patients without it

**Fig. 3:**
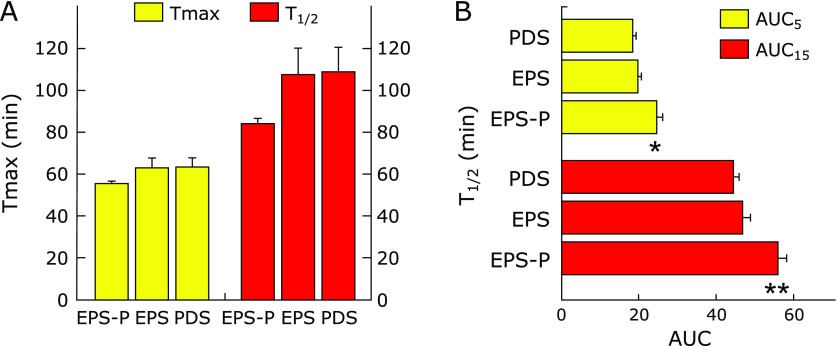
Comparison of gastric motility between EPS patients with pancreatic enzyme abnormalities and EPS patients without it. (A) There are no significant differences in Tmax and T_1/2_ values among EPS patients with pancreatic enzyme abnormalities, EPS patients without it and PDS patients. (B) AUC_5_ and AUC_15_ values in EPS patients with pancreatic enzyme abnormalities were significantly disturbed compared to those in EPS patients without pancreatic enzyme abnormalities. *p = 0.002, vs EPS patients without pancreatic enzyme abnormalities. **p = 0.001, vs EPS patients without pancreatic enzyme abnormalities.

To clarify whether gastric emptying in EPS patients with pancreatic enzyme abnormalities differs from that in EPS patients without it, we measured Tmax and T_1/2_ values for the two distinct groups. No statistically significant difference was found for Tmax and T_1/2_ values among EPS patients with pancreatic enzyme abnormalities, EPS patients without it and PDS patients (Fig. 3A). Then, to determine whether the early phase of gastric emptying in EPS patients with pancreatic enzyme abnormalities differs from that in EPS patients without it, we measured AUC_5_ and AUC_15_ values in each group. Interestingly, AUC_5 _and AUC_15 _values (24.85 ± 1.31 and 56.11 ± 2.51, respectively) in EPS patients with pancreatic enzyme abnormalities were significantly (*p* = 0.002 and *p* = 0.001, respectively) higher compared to those (19.75 ± 1.01 and 47.02 ± 1.99, respectively) in EPS patients without it (Fig. 3B). AUC_5_ and AUC_15_ values in EPS patients with pancreatic enzyme abnormalities were also significantly (*p* = 0.005 and *p* = 0.001, respectively) higher compared to those in PDS patients (Fig. 3B).

### The scores of endosonography in EPS patients with pancreatic enzyme abnormalities

**Fig. 4:**
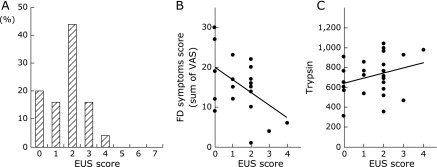
Number of abnormal endosonographic findings and the relationship between endosonographic findings and sum of VAS for FD symptoms and trypsin levels in EPS patients with pancreatic enzyme abnormalities. (A) Number of abnormal endosonographic findings in EPS patients with pancreatic enzyme abnormalities. (B) The relationship between number of abnormal endosonographic findings and sum of VAS of FD symptoms including epigastric pain, early satiety and postprandial abdominal fullness in the EPS patients with pancreatic enzyme abnormalities. There was a negatively significant (*p* = 0.014) relationship between the number of abnormal endosonographic findings and sum of VAS for FD symptoms in the EPS patients with pancreatic enzyme abnormalities. (C) The relationship between number of abnormal endosonographic findings and trypsin levels in the EPS patients with pancreatic enzyme abnormalities. There was not significant (*p* = 0.184, R = 0.281) relationship between the number of abnormal endosonographic findings and trypsin levels in the EPS patients with pancreatic enzyme abnormalities.

In this study, most of patients with major features such as pancreatic stones and pancreatic calcification who could have been diagnosed by US or CT were excluded and those who had only minor features were included. Endosonography revealed that 16/25 (64%) of EPS patients with pancreatic enzyme abnormalities had concomitant early chronic pancreatitis. Among this cohort of patients, four were diagnosed with score 1; whereas, 11 patients were diagnosed with score 2, four with score 3, and one with score 4 (Fig. 4A). To clarify whether severity of early chronic pancreatitis associates with FD symptoms including epigastric pain, we compared the score for each case of early chronic pancreatitis identified during endosonography with sum of VAS for FD symptoms including epigastric pain, early satiety and postprandial abdominal fullness in the EPS patients with pancreatic enzyme abnormalities. There was a significant (*p* = 0.014) negative correlation between the grade of EUS features in early chronic pancreatitis and FD symptoms (Fig. 4B) and the trypsin levels in EPS patients with pancreatic enzyme abnormalities did not significantly (*p* = 0.184, R = 0.281) associate with the grade of EUS features in early chronic pancreatitis (Fig. 4C).

## Discussion

The major findings of this study are; 1) the ratio of female patients in EPS patients with pancreatic enzyme abnormalities was significantly higher compared to that in EPS patients without it; 2) Physical quality of life in the EPS patients with pancreatic enzyme abnormalities was significantly disturbed compared to those in EPS patients without it and PDS patients; 3) EPS patients with pancreatic enzyme abnormalities were significantly associated with rapid early phase of gastric emptying, and 4) 64% of EPS patients with pancreatic enzyme abnormalities were diagnosed with early chronic pancreatitis proposed by Japan Pancreas Society using endosonography.

In this study, we aimed to determine whether the characteristics of EPS patients with concomitant pancreatic enzyme abnormalities differ from those of EPS patients without it. We first demonstrate that the ratio of female in EPS patients with pancreatic enzyme abnormalities are significantly higher compared to that in male. Sex appears to be more clearly associated with EUS-related features of chronic pancreatitis than age does. Yusoff *et al.*^(39)^ have reported that male sex was an independent predictor of chronic pancreatitis which was linked to accumulation of smoking and alcohol consumption in male sex, whereas patient age did not correlate with imaging findings. In addition, since EUS features of early chronic pancreatitis in Japan was exclude major A criteria in Rosemont criteria,^(40)^ male sex in advanced chronic pancreatitis may not be enrolled in EPS patients with pancreatic enzyme abnormalities using EUS. Further studies will be needed to clarify why EPS patients with pancreatic enzyme abnormalities were significantly correlated with female sex.

In this study, we compared gastric motility in EPS patients with pancreatic enzyme abnormalities with without it. Then, in our study, there was no significant differences in gastric emptying such as Tmax and T_1/2_ values between EPS patients with pancreatic enzyme abnormalities and the patients without it. There have been conflicting reports regarding gastric emptying in patients with pancreatic dysfunction.^(41–43)^ Gastric emptying has been shown to be accelerated in patients with pancreatic insufficiency^(41,42)^ and delayed in those without insufficiency.^(43)^ Interestingly, in our study, the early phase of gastric emptying in EPS patients with pancreatic enzyme abnormalities was significantly disturbed compared to that in EPS patients without pancreatic enzyme abnormalities. Kusano *et al.*^(35)^ and our previous studies^(44,45)^ have shown that FD can be attributed to either rapid or delayed gastric emptying. In addition, digestion of fat to liberate free fatty acids is essential to slow gastric emptying. Pilichiewicz *et al.*^(46)^ have reported that inhibition of fat digestion by lipase inhibitor, orlistfat, induced rapid gastric emptying. Considering our data and previous reports, disturbance of digestion of fat in EPS patients with pancreatic enzyme abnormalities may be associated with rapid early phase gastric emptying. Further studies will be warranted to investigate whether supplementation of a particular pancreatic enzyme can improve disturbed early phase of gastric emptying.

Currently, EUS may be a useful examination to identify subtle pancreatic abnormalities that may be missed by US and CT, because the close proximity of the high-frequency transducer to the pancreas may allow detection of slight pathological changes in the pancreatic ducts and parenchyma.^(21)^ There is a lack of endosonographic data available that associate pancreatic enzyme abnormalities with clinical symptoms, gastric motility and levels of the various pancreatic enzymes in patients with concomitant functional dyspepsia. Sahai *et al.*^(17)^ have reported that the mean number of endosonographic abnormalities was higher in dyspeptic patients than in control patients. Although 58/59 PDS patients have no pancreatic dysfunction, we have first reported that most of EPS patients with pancreatic enzyme abnormalities exhibited low score for early chronic pancreatitis using endosonography. In this study, 64% of EPS patients with pancreatic enzyme abnormalities had early chronic pancreatitis proposed by Japan Pancreas Society. Since EUS characteristics of early chronic pancreatitis in Japan was exclude major A factors such as hyperechoic foci with shadowing and MPD calcification, the majority of EPS patients with pancreatic enzyme abnormalities had early chronic pancreatitis with scores lower than 3.0. Recent studies have shown that the total number of EUS criteria correlates with the severity of pancreatographic changes and with reduction in secretin-stimulated duodenal biocarbonate.^(47,48)^ Sahai *et al.*^(17)^ have also reported that patients with dyspepsia without pancreatic enzyme abnormalities exhibited mild abnormalities in pancreatic ducts and parenchyma using EUS. However, in our data, severity of FD symptoms in EPS patients with pancreatic enzyme abnormalities were negatively significantly associated with the score of EUS features. In contrast, the score of EUS features was not associated with trypsin levels. Therefore, further studies are warranted to determine the correlation between EUS abnormalities and pancreatic enzyme abnormalities, clinical symptoms and gastric motility in these patients.

This study was carried out as a retrospective study in a single center with a limited number of patients. Therefore, a prospective, randomized, multicenter study is required to confirm these findings. Within these limitation, in this study, EPS patients with pancreatic enzyme abnormalities had significantly higher postprandial abdominal fullness scores and significantly lower physical component summary (PCS) scores than EPS patients without pancreatic enzyme abnormalities. Early phase of gastric emptying was significantly impaired in EPS patients with pancreatic enzyme abnormalities compared to that in EPS patients without it. Further studies are warranted to clarify how severity of clinical symptoms, abnormality of pancreatic enzymes and disturbance of early phase of gastric emptying associate with the promotion of early chronic pancreatitis.

## Figures and Tables

**Table 1:** Characteristics of PDS patients, EPS patients with pancreatic dysfunction and EPS patients without it

	EPS-P	EPS	PDS
	(*n* = 41)	(*n* = 42)	(*n* = 59)
Age	26–86	26–85	18–85
Sex (F/M)	F34/M7	F20/M22	F24/M35
GSRS	2.61 ± 0.17	2.64 ± 0.14	2.48 ± 0.17
HP positivity	14.3%	16%	15.2%

**Table 2:** Comparison of PSQI, SF-8 and STAI among EPS patients with pancreatic dysfunction, EPS patients without it and PDS patients

	EPS-P	EPS	PDS
PSQI	4.77 ± 0.48	6.95 ± 0.58*	6.43 ± 2.68**
PCS	37.9 ± 2.61	44.5 ± 1.06^†^	46.1 ± 3.78^††^
MCS	40.5 ± 2.46	45.3 ± 1.19	46.8 ± 3.84^ϕ^
STAI-state	54.0 ± 4.79	50.9 ± 3.93	44.3 ± 3.59
STAI-trait	47.4 ± 5.03	46.6 ± 4.41	39.9 ± 2.32

ESP-P: epigastric pain syndrome with pancreatic dysfunction. *p = 0.005, vs EPS-P; **p = 0.016, vs EPS-P; ^†^*p* = 0.024, vs EPS-P; ^††^*p* = 0.004, vs EPS-P; ^ϕ^*p* = 0.02, vs EPS-P.
